# Does the esv3587290 Copy Number Variation in the *VANGL1* Gene Differ as a Genetic Factor for Developing Nephritis in Mexican Childhood-Onset Systemic Lupus Erythematosus Patients?

**DOI:** 10.3390/children11060712

**Published:** 2024-06-10

**Authors:** Miguel Angel Alcántara-Ortigoza, Ana Luisa Rodríguez-Lozano, Bernardette Estandía-Ortega, Ariadna González-del Angel, Luisa Díaz-García, Francisco Eduardo Rivas-Larrauri, Ruth Guadalupe Nájera-Velázquez

**Affiliations:** 1 Laboratorio de Biología Molecular, Subdirección de Investigación Médica, Instituto Nacional de Pediatría, Ciudad de México 04530, Mexico; bernsestandia@yahoo.com.mx (B.E.-O.); ariadnagonzalezdelangel@gmail.com (A.G.-d.A.); 2 Servicio de Inmunología, Instituto Nacional de Pediatría, Ciudad de México 04530, Mexico; anarlozano@yahoo.com.mx (A.L.R.-L.); rivaslarrauri@yahoo.com (F.E.R.-L.); ruth12.nv@gmail.com (R.G.N.-V.); 3 Departamento de Metodología de la Investigación, Subdirección de Investigación Médica, Instituto Nacional de Pediatría, Ciudad de México 04530, Mexico; luisadiazg@gmail.com

**Keywords:** childhood-onset systemic lupus erythematosus, lupus nephritis, *VANGL1* gene, DNA copy number variation, Mexican population

## Abstract

A ~3-kb deletion-type DNA copy number variation (CNV, esv3587290) located at intron 7 of the *VANGL1* gene (1p13.1, MIM*610132) has been proposed as a genetic factor in lupus nephritis (LN) development in adult systemic lupus erythematosus (SLE) patients across European-descent populations, but its replication in other ethnicities has been inconsistent and its association with LN in childhood-onset SLE (cSLE) remains unknown. Here, we performed an exploratory association study in a sample of 66 unrelated cSLE Mexican patients (11 males, 55 females; ages 7.8 to 18.6 years). Two stratified groups were compared: cSLE patients with (N = 39) or without (N = 27) LN, as diagnosed by renal biopsy (N = 17), proteinuria (N = 33), urinary protein–creatinine ratio > 0.2 (N = 34), and erythrocyturia and/or granular casts in urinary sediment (N = 16). For esv3587290 CNV genotyping, we performed an end-point PCR assay with breakpoint confirmation using Sanger sequencing. We also determined the allelic frequencies of the esv3587290 CNV in 181 deidentified ethnically matched individuals (reference group). The obtained genotypes were tested for Hardy–Weinberg equilibrium using the χ^2^ test. Associations between LN and esv3587290 CNV were tested by calculating the odds ratio (OR) and using Pearson’s χ^2^ tests, with a 95% confidence interval and *p* ≤ 0.05. The esv3587290 CNV allele (OR 0.108, 95% CI 0.034–0.33, *p* = 0.0003) and the heterozygous genotype (OR 0.04, 95% CI 0.119–0.9811, *p* = 0.002) showed a significant protective effect against LN development. Finally, we characterized the precise breakpoint of the esv3587290 CNV to be NG_016548.1(NM_138959.3):c.1314+1339_1315-897del in our population. This report supports the notion that a broad genetic heterogeneity underlies the susceptibility for developing LN.

## 1. Introduction

Systemic lupus erythematosus (SLE) is a chronic, multisystem, autoimmune, and inflammatory disease that is most often of unknown cause. It is characterized by the production of multiple autoantibodies, especially against nuclear components (e.g., double-stranded DNA, dsDNA); this can generate inflammatory-mediated effects in any organ and/or system. Childhood-onset forms (cSLE, onset before age 18) represent 20% of all cases [1,2]. Lupus nephritis (LN) is generally estimated to occur 10–30% more frequently in cSLE (40–70%) than in adult forms of SLE [2,3,4]. This complication is among those with the highest impacts on the life quality and survival of SLE patients since it increases the risk of developing end-stage kidney disease (ESKD) [5]. LN shows an earlier onset and more severe clinical course in cSLE compared to adult SLE forms; additionally, it has been reported as a negative predictor of survival [4].

Identifying biomarkers for the diagnosis, prognosis, and non-invasive evaluation of renal disease activity in SLE is a rapidly evolving field [6]. However, in the clinical setting, LN diagnosis and the evaluation of renal flares still rely on the assessment of traditional serum (e.g., serum creatinine, erythrocyte sedimentation rate, complement components C3 and C4, glomerular filtration rate, anti-C1q or anti-dsDNA autoantibody titers, etc.), and urinary (urine sediment, proteinuria, albumin–creatinine ratio, etc.) biomarkers, or on the histological evaluation of renal biopsies [6,7,8].

To date, genome-wide association studies (GWASs) have identified around 100 susceptibility genes for SLE development [6,9]. The relevant genetic changes have mainly been related to single-nucleotide variations in immune-response genes (e.g., HLA-DR3 in patients of European ancestry) [9,10], along with some gene copy number variations (CNVs) in non-immune-related genes, such as *VANGL1* (1p13.1, MIM*610132) [11]. *VANGL1* is an essential gene in the establishment of planar cell polarity (PCP), and heterozygous variants have been associated with neural tube defects (MIM#182940) and caudal regression syndrome (MIM#600145), but not with human kidney disease. A GWAS performed in 55 patients with SLE, 11 with Sjogren’s syndrome, and 11 healthy controls identified three SLE patients homozygous for a *VANGL1* deletion-type CNV of ~3.17 kb (esv3587290) located at intron 7. Of these patients, two had LN. The authors furthermore evaluated the esv3587290 using the TaqMan^®^ assay in 177 SLE patients of mainly European descent. The results revealed that the deletion was significantly associated with LN (χ^2^ = 27.06, 2 d.f., *p* < 0.0001) and demonstrated a gene–dosage effect. Moreover, the esv3587290 CNV seems to be a highly prevalent allele in the Australian Aboriginal Tiwi Islander population, who present high rates of kidney disease [11]. However, in a third SLE cohort of mainly Spanish-descent patients (N = 281, χ^2^ = 2.1, 1 d.f., *p* = 0.14), this association was not replicated [11]. Indeed, a *Vangl1^−/+^* model mice showed spontaneous deposition of IgA and IgG, but not of IgM or complement, in the mesangium [11]. This led researchers to hypothesize that a deficiency of Vangl1 protein in heterozygous mice could alter the permeability of the glomerular endothelium for monomeric immunoglobulins. Despite the association of esv3587290 CNV with LN, no convincing evidence for a deleterious effect of this CNV on *VANGL1* function was achieved in this study; however, the experimental murine models also supported that Vangl1 deficiency was only associated with the development of nephritis in the *Vangl1^−/+^* mice injected with autoreactive serum, which further supports an altered glomerular endothelial permeability to autoreactive immunoglobulins. Whether this mechanism underlies an association of the esv3587290 CNV with LN development in humans remains unknown [11].

African American, Hispanic, and Asian SLE patients are at greater risk for developing and presenting more severe forms of LN compared to those from European-descent populations [10]. To our knowledge, no GWAS has yet identified any LN susceptibility locus linked to *VANGL1* or the 1p13.1 region, even in populations of European ancestry [9,10,12,13]. Furthermore, Jiang et al. failed to replicate the association of LN with the esv3587290 CNV in a cohort of predominantly Spanish-descent patients [11]. Thus, additional work is needed to test whether this genetic variation could be a risk factor in other ethnicities or clinical presentations of SLE, such as the childhood-onset form. Finally, Jiang et al. employed whole-genome sequencing (WGS) to reveal that the esv3587290 deletion-type CNV varied in size among SLE patients [11], although the authors did not report precise nucleotide-resolution breakpoints using the Human Genome Variation Society (HGVS) nomenclature [14]. This size variability could suggest that distinct mutational events may generate the CNV. Meanwhile, given its high minor allelic frequencies in different populations (0.17–0.43) [11], we cannot discard the possibility of a one-time emergence of a single common allele.

Here, we sought to determine if the *VANGL1* esv3587290 deletion-type CNV is associated with LN in a sample of Mexican cSLE patients, and to characterize the precise breakpoints of this rearrangement in our cSLE patients as well as a reference group of ethnically matched individuals.

## 2. Materials and Methods

### 2.1. Patient Selection

We enrolled a total of 66 unrelated Mexican children (11 males, 55 females; aged 7.8 to 18.6 years) who were born from Mexican parents in the central region of Mexico. These patients were diagnosed with cSLE between the years 2008 to 2022, with an average age of 11.19 ± 3.31 years, as established from the pediatric immunology service at Instituto Nacional de Pediatría, México. For inclusion, subjects were required to fulfill the criteria of the 2012 Systemic Lupus International Collaborating Clinics [15] or the 2019 European League Against Rheumatism/American College of Rheumatology Classification Criteria for Systemic Lupus Erythematosus [16]. Patients with suspected (disease onset < 5 years, parental consanguinity, or familial history of autoimmune disease in a first-degree relative) or a confirmed diagnosis of a monogenic form of cSLE were not included. Diagnosis of LN was based on the presence of proteinuria (>0.5 g/dL) or a urinary protein–creatinine (UPr–Cr) ratio > 0.2, erythrocyturia, the presence of granular casts in urinary sediment, and/or histopathological confirmation through renal biopsy.

To estimate the allelic esv3587290 CNV frequencies in our population and determine the necessary sample size, we included 181 deidentified genomic DNA samples from unrelated Mexican newborns. The DNA was obtained from residual dried blood spot (DBS) samples analyzed for newborn screening (reference group).

### 2.2. Genotyping of the esv3587290 CNV

Buccal cell swabs obtained from the cSLE patients, and DBS samples from the reference group, were processed by using the standard salting-out method to obtain genomic DNA. We initially performed a PCR assay using forward (5′-AGGGGAGGTGATGGACCCTA-3′) and reverse (5′-CTCAGACTGTAAGCGAAGGACA-3′) primers located inside exons 7 and 8 of *VANGL1*, respectively, to identify the esv3587290 CNV. This assay generated ~6 kb and ~3 kb PCR fragments from the wild-type and esv3587290 CNV alleles, respectively. This strategy was initially applied to 16 genomic DNA samples from the reference group, and the ~3 kb PCR fragments were identified in three individuals. These ~3 kb PCR fragments were gel excised, purified, and subjected to Sanger sequencing using a “primer-walking” strategy (primers are available upon request). As the entire ~3 kb sequence revealed a single identical breakpoint in these amplicons, we designed a new set of primers (VANGL1-INT7-CNVdelB-F: 5′-TGGCTGTTTCTTGTAATATCCC-3′ and VANGL1-INT7-CNVdelB-R: 5′-CCGACATGGTAAGCAAGC-3′) to amplify a shorter fragment (521 bp) encompassing the breakpoint boundaries of the esv3587290 CNV. To detect the non-deleted *VANGL1* allele, we designed a set of primers (VANGL1-INT7A-F: 5′-ACTGATTGTCTGTTGATGCACATTT-3′ and VANGL1-INT7A-R: 5′-CACCCCCTAGGAGGGCAAT-3′) to amplify an internal region of intron 7 (357 bp) that is absent from the esv3587290 CNV sequence. These two mutually exclusive amplicons were generated by two separate monoplex end-point PCR assays (PCR conditions are available upon request) and resolved by agarose gel electrophoresis. Allelic and genotypic frequencies were obtained by direct counting in the cSLE patient and reference group samples as follows: wild-type homozygous, 357 bp fragment; heterozygous, 521 and 357 bp fragments; and esv3587290 CNV homozygous, 521 bp fragment. All the 521 bp esv3587290 CNV-derived amplicons from the cSLE patients and reference group individuals were subjected to direct automated Sanger sequencing and further alignment (Program Chromas Pro Version 2.1.10, Technelysium Pty Ltd., South Brisbane, QLD, Australia) with the gene (NG_016548.1) and Vang-like protein 1 isoform 1 (NM_138959.3) reference sequences to determine the precise breakpoints. The rearrangement was reported according to HGVS nomenclature (https://hgvs-nomenclature.org/stable/; accessed on 11 March 2024) [14].

### 2.3. Statistical Analysis

The sample size was calculated by applying a formula to find differences between two proportions, using reference-group allelic frequencies for the wild-type (0.765) and esv3587290 CNV (0.235) *VANGL1* (Table 1), employing a confidence level of 99% and a power of 95%, and assuming a 10% loss to follow-up. From this, 33 individuals had to be included for each cSLE group (with and without LN).

The *VANGL1* genotypes obtained from the reference samples and cSLE patients were tested for the Hardy–Weinberg equilibrium (HWE) using the χ^2^ test. Associations between the presence of LN and the presence of the esv3587290 CNV were examined by using odds ratio (OR) calculations and Pearson’s χ^2^ test, employing a confidence interval of 95% and a significance threshold of *p* ≤ 0.05. These calculations were performed using the IBM^®^ SPSS^®^ Statistics software Version 25.0.

This study protocol was revised and approved by the Institutional Review Research, Biosecurity, and Ethics Committees of the National Institute of Pediatrics, Mexico (Registry 2022/030; approval date 20 June 2022). We conducted this study according to the guidelines of the Declaration of Helsinki.

## 3. Results

LN was present in 39 of 66 (59.1%) patients at the moment of their inclusion in this study. Seventeen patients had undergone renal biopsy, and histology was available in all of them (five with class II, two with class III, eight with class IV, and two with class V of LN stratification), while thirty-three patients had 24 h proteinuria > 0.5 g/dL, thirty-four had a UPr–Cr ratio > 0.2, and sixteen had erythrocyturia and/or granular casts in the urinary sediment. Most of the patients without LN (N = 20/27) had a medical follow-up of more than 2 years, while the remaining seven underwent their last immunologist-conducted evaluation between 19 and 23 months after cSLE diagnosis.

The allelic and genotypic frequencies of the esv3587290 CNV in cSLE patients and reference samples are shown in Table 1. The proportions of genotypes in both groups were consistent with HWE, as assessed by using the χ^2^ test. The esv3587290 CNV alleles and cSLE patient genotypes showed significant associations with the presence of LN, as assessed using the χ^2^ test. More specifically, the esv3587290 CNV (OR 0.108, 95% CI 0.034–0.33, *p* = 0.0003) and the heterozygous genotype (OR 0.04, 95% CI 0.119–0.9811, *p* = 0.002) were observed to show a protective effect on the development of LN. The relatively low number of cSLE patients carrying a homozygous esv3587290 CNV genotype in the groups with and without LN precluded χ^2^ testing for this comparison (Table 2).

**Figure 1:**
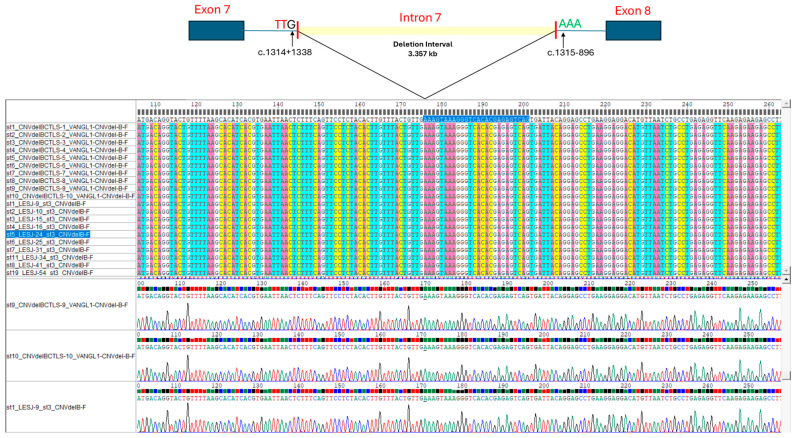
Schematic of the esv3587290 CNV deletion interval in intron 7 of *VANGL1* and partial forward sequence alignments of the 521 bp PCR fragments, revealing the identical breakpoint of the esv3587290 CNV deletion in selected cSLE patients (N = 10) and reference samples (DBS, N = 10). The same breakpoint was observed for the 38 and 85 esv3587290 CNV alleles (Table 1) identified in the cSLE patients and reference samples, respectively, leading us to establish this gene rearrangement as NG_016548.1(NM_138959.3):c.1314+1339_1315-897del.

The Sanger sequencing results of all the 521 bp PCR fragments derived from the esv3587290 CNV identified in the reference and cSLE samples showed an identical breakpoint, wherein deletion of 3357 bp eliminates nearly 60% of the intron 7 sequence (5591 bp) of *VANGL1* (Figure 1). We therefore designated this common deletion as NG_016548.1(NM_138959.3):c.1314+1339_1315-897del, NC_000001.10:g.116229487_116232843del (GRCh37), or NC_000001.11:g.115686866_115690222del (GRCh38) according to the HGVS nomenclature. This variant was submitted to the Leiden Open Variation Database (LOVD) of *VANGL1* gene (https://databases.lovd.nl/shared/variants/0000918418#00025811; accessed on 15 February 2023).

## 4. Discussion

Replication studies are mainly intended to confirm genetic associations discovered through GWAS, such as that between esv3587290 CNV and SLE-related LN [11]. These studies are needed to accumulate convincing statistical evidence that supports the association and rules out spurious findings due to uncontrolled biases [17]. To the best of our knowledge, this is the first work to explore the possible association of the esv3587290 CNV with LN in a non-European population, as previously recommended [11]. Here, we assessed an SLE population of Mexican descent, who are among the ethnicities considered to have a high risk of developing LN [18]. We further focused on a clinical form of SLE different than that previously studied in this context, namely childhood-SLE, for which LN is considered to be more prevalent and severe than in the adult form of SLE [4].

A few genetic markers have been associated with LN in Mexican populations, including *SPP1* (MIM*166490) in adult SLE [allele T of rs1126616 OR 2.0 (95% CI 1.26–3.16), *p* = 0.003 and TT genotype under the recessive model OR 2.76 (95% CI 1.31–5.82), *p* = 0.011] [19] and *NFE2L2* (formerly *NRF2*; MIM*600492) in cSLE [heterozygous A/G rs35652124 genotype OR = 1.81 (95% CI 1.04–3.12), *p* = 0.032) [20]. Meanwhile, other markers of LN susceptibility previously identified in European-descent populations (e.g., *PDCD1*, MIM*600244) [21] or murine models (CNVs of *TLR7*, MIM*300365) [22] have failed to show any significant association with LN in cSLE patients from Mexican populations [23,24]. These previous observations, together with our present finding that esv3587290 CNV appears to protect against the development of LN in this group of Mexican patients with cSLE (Table 2), could support the idea that LN exhibits broad genetic and phenotypic heterogeneity. Our results may also agree with the lack of an evident association between LN and *VANGL1*, the 1p13.1 region, or PCP pathways in a GWAS performed in Hispanic, European, African American, and Asian patients [13]. We further believe that our findings and the inability of Jiang et al. to replicate their association in the third cohort suggest that, in contrast to the previous proposal [11], esv3587290 CNV genotyping is not a viable strategy for LN risk stratification, at least for non-European SLE populations and cSLE patients.

We further characterized the precise breakpoint of the esv3587290 CNV in our study population. Whilst Jiang et al. found that this deletion varied in size when assessed using a WGS strategy [11], our Sanger sequencing approach revealed an identical deletion event in all individuals carrying one or two esv3587290 CNV copies (Figure 1). We speculate that these discrepancies may reflect different origins of the esv3587290 CNV among diverse populations, or they may be related to methodological issues. The former could imply that there is a “hot-spot” for mutational events leading to distinct rearrangements, as occur for some monogenic traits (i.e., gross deletions in the *DMD* gene). Alternatively, some single mutational events may occur and may even be associated with a founder effect [25], as appears to be the case for the esv3587290 CNV in our Mexican population. Further haplotypic analysis is warranted to support the latter hypothesis. Regarding the potential impact of methodologic issues, we note that the short-read WGS strategy is generally intended to approximately localize the breakpoints of a gross genomic structural rearrangement (e.g., a deletion-type CNV); to reach a nucleotide-level resolution, it would be necessary to apply long-range PCR and Sanger sequencing [26,27], as performed herein. Given this, we propose that it would be desirable to determine the precise nucleotide-level rearrangements of the esv3587290 CNV in other populations. In the PCR-based Sanger sequencing strategy, we used the forward and reverse primers designed to anneal ~250 bp away from the breakpoints estimated by WGS to amplify the esv3587290 CNV [11]. There remains some possibility that allelic drop-out may have occurred due to the non-amplification of alleles bearing different breakpoints. However, we believe that this is unlikely due to the lack of departure from HWE in both study groups, along with the similarity between our allelic frequencies (Table 1) and those previously reported for esv3587290 CNV in Latino populations (~0.3) [11].

Although mis-spliced mRNA species of *VANGL1* lacking exon 2 were identified in peripheral blood mononuclear cells from two of the homozygous esv3587290 CNV SLE patients, whether the esv3587290 CNV has any effect on *VANGL1* function at the kidney remains to be determined [11]. Such a finding would support the idea that this CNV plays a role in genetic susceptibility to the development of LN in humans. Interestingly, a predicted enhancer element is located inside exon 8 (encoding the 3′UTR) of *VANGL1* (https://www.ncbi.nlm.nih.gov/gene/81839; accessed on 11 March 2024; chr1:116238833-116239332; GRCh37/hg19 assembly coordinates). It would be interesting to determine if the neighboring esv3587290 CNV exerts an effect on the functionality of this predicted enhancer element in human kidney tissues.

### Limitations of the Study

Nonetheless, our study had several limitations: It is possible that our study sample was under-representative or phenotypically heterogeneous, given the lack of renal biopsy-proven LN in most of our patients (N = 22/39 LN cSLE patients, 56.4%), which is still considered the diagnostic gold standard [7]. Obtaining such information could allow us to perform an association analysis stratified by histopathological classes, as previously recommended [11]. Also, we did not perform any ancestry analysis to determine if the reference group and cSLE patients were admixed; thus, a possibility of bias by population stratification cannot be excluded. The low number of male cSLE patients (nine with LN and two without LN) did not allow us to perform stratification analysis by gender, so this aspect should be addressed in the future. It is important to note that the above-described aspects were also not considered by Jiang et al. [11]. Genotyping errors could have biased our analysis, as we did not use a second molecular technique to validate our PCR-based genotyping assay. However, this seems unlikely given the lack of esv3587290 CNV departure from HWE in the reference group and cSLE patients, along with the similarity between the observed allelic frequencies (Table 1) and those previously reported in Latino populations [11]. Finally, the cross-sectional nature of this study precluded us from determining whether the included cSLE patients without LN could later develop nephritis, particularly in those seven cSLE patients with normal kidney function who did not complete the two years of follow-up, adding another potential confounding factor. The estimated risk of developing LN in these patients is expected to be 7% [28].

## 5. Conclusions

The esv3587290 CNV of the *VANGL1* gene was not associated with the development of LN in a sample of Mexican cSLE patients; rather, this CNV seems to be a genetic protective factor. Further replication studies on the esv3587290 CNV in other ethnicities and clinical forms of SLE are warranted to define its role as a genetic factor in the development of LN. The esv3587290 CNV seems to be a unique 3357 bp deletion that may have originated from a single mutational event in our Mexican population.

## Figures and Tables

**Table 1:** Allelic and genotypic frequencies of esv3587290 CNV in cSLE patients and reference samples.

*VANGL1* Alleles	Total cSLE Patients (N = 66, 132 Alleles)	Reference Group (DBS Samples) (N = 181, 362 Alleles)
Wild-type allele	0.712	0.765
esv3587290 CNV- deletion-type allele	0.288	0.235
***VANGL1*** **Genotype**	**Total cSLE Patients** **(N = 66 Genotypes)**	**Reference Group** **(DBS Samples)** **(N = 181 Genotypes)**
Wild-type homozygous	48.5% (N = 32)	58% (N = 105)
Heterozygous	45.5% (N = 30)	37% (N = 67)
Homozygous esv3587290	6% (N = 4)	5% (N = 9)

Abbreviations: cSLE: childhood-onset systemic lupus erythematosus; CNV: copy number variation; DBS: residual anonymized dried blood spots from Mexican unrelated neonates subjected to newborn screening program.

**Table 2:** Association results and *VANGL1* allelic and genotypic frequencies among cSLE patients with or without LN.

*VANGL1* Alleles	Allelic Frequencies cSLE Patients with LN (N = 39, 78 Alleles)	Allelic Frequencies cSLE Patients without LN (N = 27, 54 Alleles)	χ^2^/ OR (95% CI) ^1^	*p*-Value
Wild-type allele	0.769	0.630	χ^2^ = 16.41 OR = 0.108 (0.034–0.33)	*p* = 0.000025 *p* = 0.0003
esv3587290 CNV deletion-type allele	0.231	0.370		
***VANGL1*** **Genotypes**	**Genotypic Frequencies** **cSLE Patients** **with LN** **(N = 39 Genotypes)**	**Genotypic Frequencies** **cSLE Patients** **without LN** **(N = 27 Genotypes)**	**χ^2^/** **OR (95% CI)**	***p*-Value**
Wild-type homozygous	59% (N = 23)	33.3% (N = 9)		
Heterozygous esv3587290	35.9% (N = 14)	59.3% (N = 16)	χ^2^ = 4.089 ^2^ OR = 0.04 ^2^ (0.119–0.9811)	*p* = 0.02 *p* = 0.002
Homozygous esv3587290	5.1% (N = 2)	7.4% (N = 2)	χ^2^ = not calculable ^3^ OR = not calculable ^3^	*p* = not calculable *p* = not calculable

^1^ The presence of LN, esv3587290 alleles, and heterozygous genotypes were taken as the reference categories for the OR calculations. ^2^ Wild-type homozygous vs. heterozygous for esv3587290; ^3^ Wild-type homozygous vs. homozygous for esv3587290. Abbreviations: CI: confidence interval; cSLE: childhood-onset systemic lupus erythematosus; CNV: copy number variation; OR: odds ratio.

## Data Availability

Publicly available datasets were analyzed in this study. These data can be found in the LOVD v.3.0—Leiden Open Variation Database: https://www.lovd.nl/, accessed on 11 March 2024; OMIM: https://www.omim.org/, accessed on 11 March 2024; NCBI: https://www.ncbi.nlm.nih.gov/gene, accessed on 2 April 2024. The data presented in this study are available on reasonable request from the corresponding author. All the herein reported clinically relevant genetic variants along with the available deidentified phenotypic data were submitted to the publicly available LOVD v.3.0—Leiden Open Variation Database (https://databases.lovd.nl/shared/variants/0000918418#00025811, accessed on 11 March 2024).
